# Authorization of the first COVID-19 emergency vaccines: The matters arising

**DOI:** 10.17179/excli2021-3384

**Published:** 2021-03-15

**Authors:** Kamoru A. Adedokun, Ramat T. Kamorudeen, Ibrahim O. Bello

**Affiliations:** 1 Department of Oral Pathology, DUH, King Saud University Medical City, Riyadh, Kingdom of Saudi Arabia; 2 Children Welfare Unit, Osun State Hospital Management Board, Asubiaro, Osogbo, Osun State, Nigeria; 3 Department of Public Health, University of South Wales, UK; 4 Department of Medical Laboratory Science, Nigerian Navy School of Health Sciences, Offa, Kwara State, Nigeria

## ⁯

***Dear Editor, ***

Recently, the United States Food and Drug Administration (FDA) gave the Pfizer/BioNTech and Moderna coronavirus disease 2019 (COVID-19) vaccines emergency use authorizations (EUAs). The vaccines contain synthetic mRNA vaccine candidates for COVID-19 (ClinicalTrials.gov/NCT04368728; BioNTech SE, Pfizer, 2020[1]). According to the FDA, when medical products meant to treat, detect or prevent COVID-19 are unavailable or inadequate, the regulatory body may authorize the existing unapproved alternatives in emergencies (FDA, 2020[4]). Invariably, vaccines with EUA cannot be treated the same as many established vaccines such as diphtheria, tetanus, and whooping cough (pertussis) (DTaP), among others, that had been studied for many years. Therefore, emergency vaccines that are yet to complete their trials and without adequate follow-up time still have many unanswered questions. Understanding potential risks of the unknown and that, “efficacy” does not literally mean “effectiveness” in the clinic, this report seeks answers that would be at an advantage of human safety in addressing the current global threats of COVID-19 disease.

Pfizer is a giant pharmaceutical company that joined a German company, BioNTech and employed synthetic mRNA technology to induce an immune response, in the same way with Moderna's, an American company. The Pfizer/BioNTech vaccines consist of two biologicals - BNT162b1 and BNT162b2. Both are lipid nanoparticle-formulated, nucleoside-modified RNA vaccine candidates. The former encodes a trimeric SARS-CoV-2 receptor-binding domain (RBD) while the latter encodes a membrane-anchored SARS-CoV-2 full-length spike (Walsh et al., 2020[11]). According to the clinical trial information, the estimated primary date of tryout completion of the Pfizer/BioNTech vaccine will be due by July 30, 2021, whereas the estimated final study completion is January 27, 2023 (ClinicalTrials.gov/NCT04368728; BioNTech SE, Pfizer, 2020[1]). Moderna, on the other hand, is a biotech company situated in Cambridge, Massachusetts. The Moderna employs mRNA-173 vaccine for symptomatic COVID-19 patients in a clinical trial (ClinicalTrials.gov/ NCT04470427; Moderna TX, Inc). Although the mechanism is similar with the Pfizer-BioNTech vaccine, they have little differences in terms of efficacies (Pfizer/BioNTech: 95 %, Moderna: 94.5 %), and storage specification (Pfizer/BioNTech: -80 to -60 °C; Moderna: -25 to -15 °C), among few others (FDA, 2020[4]; Pfizer, BioNTech, 2020[9]). The vaccines are said to be safe, tolerable, and efficacious against COVID-19 (ClinicalTrials.gov/NCT04368728; BioNTech SE, Pfizer, 2020[1]; FDA, 2020[4]). However, there is a paucity of clinical data to suggest whether any potential unknowns or long-term adverse effects would not occur. Even though scientists keep monitoring any unforeseen events that may unfold at any point in time, the vaccines still have many questions left unanswered.

### For how long will the vaccines confer immunity, with or without booster shots?

While the efficacy report has limited clinical data to suggest possible effectiveness over time, scientists would like to have deep knowledge about the effectiveness of the vaccines. Will the vaccines confer long-term immunity? Will they require timely booster shots, and at what time intervals, if yes? From the 2/3 phases of the clinical trials of placebo-controlled randomized studies sponsored by Pfizer/BioNTech and Moderna, the studies commenced in the mid of 2020 and were authorized for clinical use before the year ended. Generally, vaccine development and testing takes about 10-15 years long in a complex process and often involves many stages such as exploratory, preclinical and clinical studies. The first steps involve laboratory and animal studies. They are further categorized into exploratory and pre-clinical stages and are carried out before clinical studies on human volunteers. The exploratory phase often lasts for 2-4 years and involves basic laboratory research to identify natural or synthetic antigens that could be useful as a vaccine candidate to prevent a particular disease. Pre-clinical studies, on the other hand, usually last 1-2 years and may involve an *in vitro* procedure (cell or tissue culture system) or animal studies using mice, rats, guinea pigs, Rhesus macaque (*Macaca mulatta*), and calves, among others. The goal is to understand a typical immune response expected in humans by first testing the vaccine candidate in animal models for appropriate immunogenicity. At this stage, a perfect method of administration, as well as a maximum safe starting dose is properly investigated and documented. This does not only offer a good opportunity to improve on the effectiveness of that vaccine candidate but also ensures it is safe for human use.

Since the FDA authorized the Pfizer and Moderna vaccines, the biomedical ethicists have questioned the skipping of animal trials. Professor Jonathan, a biomedical ethicist at the McGill University, Canada opined, “Outbreaks and national emergencies often create pressure to suspend rights, standards and/or normal rules of ethical conduct. Often our decision to do so seems unwise in retrospect,” (Boodman, 2020[2]). However, according to Moderna Inc's Chief Medical Officer, Tal Zaks stated, “I don't think proving this in an animal model is on the critical path to getting this to a clinical trial” (Boodman, 2020[2]). Similarly, many experts believe the vaccines followed the normal protocol considering the pressing need for a vaccine in a threatening pandemic situation. The director for the International Vaccine Access Center at Johns Hopkins University, Dr Williams Moss noted, “They overlapped preclinical studies with the early phases of the trials. One of the reasons we are even talking about vaccines now just 10 months later is that some of the phases in which vaccine development normally occurs were overlapped rather than done sequentially." One of the experts' opinions is that Pfizer and Moderna were approved to investigate the vaccines on mice and macaques while they were on Phase 1 human trials, concurrently. 

However, whether the processes of the vaccine development were skipped or overlapped, it is important to note that over 30,000 participants have been involved in the clinical trial sponsored by Moderna. Similarly, over 40,000 participants have been involved in Pfizer-BioNtech vaccine trials in six different countries (Pfizer Inc., 2020[8]). Logistically, the participants' recruitment was not all of a sudden. It shows that the follow-up time after the first trial shots of the biologicals was less than five months following the participants' recruitment.

However, the vaccines are reported to be effective without any clinical concerns, nevertheless, the short follow-up time remains a cause for concern in determining the effective duration the vaccines could proffer immunity. The two biologicals of Pfizer-BioNtech are currently under phases 2/3. Meanwhile, multiple vaccines are expected for approval across the globe with detailed information of their efficacies and the time interval they are expected to induce immunity (The Lancet Microbe, 2021[6]). Until now, there is still a paucity of clinical data regarding the effectiveness of the vaccines. Even though the efficacy reports have been published, it is not understood whether the vaccines would confer life immunity or at some points requiring booster shots. 

### Will the vaccines have any impact on the transmission of SARS-CoV-2?

The target goal of the available vaccines is personal protection from the SARS-CoV-2 infection. However, there is little information about whether the vaccines would have a significant impact on the current spate of SARS-CoV-2 transmission. In other words, there is little knowledge to suggest whether the emergency vaccines would limit the current rapid transmission over time, especially to the yet-to-be vaccinated population. Understanding the logistic reason and economic situation in many countries, particularly in the low-income settings where the vaccines may not be adequately sufficient or available, several people would not be vaccinated so soon. Consequently, in the mixed populations of both vaccinated and unvaccinated, it is not currently understood whether the latter is safe from SARS-CoV-2 transmission. At present, there is limited clinical data to address public safety regarding the risk of exposure to individuals that have received shots- should the vaccinated populations continue the masking in the ongoing COVID-19 global transmission threat or they pose no threat in reality? 

### Will the vaccines be effective and safe for specific groups excluded during clinical trials?

Another area of safety concern was the exclusion of some specific groups in clinical trials. Individuals with recent SARS-CoV-2 infection, asymptomatics, immunocompromised, pediatrics, pregnant and breastfeeding women, among others, were excluded during efficacy testing. Will the vaccines be effective for these groups of the population in the clinic without any adverse effects? Until now, no clear information on the potential outcomes regarding vaccinating these groups of individuals. Also, in their eligibility criteria (exclusion list), some groups of the individual with high-risk factors for COVID-19 such as diabetics, asthmatics, hypertensives, obese and immunodeficient individuals, among others are excluded from the ongoing clinical trials (ClinicalTrials.gov/NCT04368728; BioNTech SE, Pfizer, 2020[1]). However, the risk of SARS-CoV-2 infection is worth counterpoised, whether any of the excluded groups would be fit for the vaccines or potential complication of the unknown could emerge when vaccinated is an important safety measure to be weighed in counterbalance.

### Will the vaccines be effective against the new and future mutant strains of SARS-CoV-2?

Currently, there is global concern about the sudden emergence of several SARS-CoV-2 variants with different mutations. Of most worrisome are the new SARS-CoV-2 strains that emerged in the fall, 2020, which are currently circulating globally. Most notable among these strains are the United Kingdom (UK) variant, South African variant and the Brazilian variant. The UK variant is known as 20I/501Y.V1, Variant of Concern 202012/01 (abbreviated VOC 202012/01) or B.1.1.7 with a large number of mutations. It is associated with higher mortality and transmissibility rates compared to any other variant. In the UK-variant lineage, the mutation occurs and affects the RBD of the spike protein at position 501. Precisely, the amino acid asparagine (N) is replaced with tyrosine (Y) shortened as N501Y (CDC, 2021[3]). The South African variant is known as 20H/501Y.V2 or B.1.351. It shares some mutations with B.1.1.7 strain. The B.1.351 lineage is also affected by multiple mutations affecting the spike protein involving K417N, E484K, and N501Y. The Brazilian variant is referred to as P.1. In the same way, the P.1 strain is also affected by multiple mutations. Akin to the UK-variant, three of the mutations in the P.1 strain affect the RBD of the spike protein (CDC, 2021[3]). Importantly, there are several potential consequences of mutations occasioned by the emerging SARS-CoV-2 variants such as increased disease transmissibility (Wu et al., 2021[13]), reduced susceptibility to therapeutics (Weisblum et al., 2020[12]), worsening disease severity, evasion of viral detection by specific diagnostic tests, and evasion of natural or vaccine-induced immunity (CDC, 2021[3]).

Now that the era of COVID-19 vaccines has begun, in connection with vaccine effectiveness, one striking question remains. Can the people at risk of the new mutant strains of SARS-CoV-2 get protection from the available vaccines? Researchers have swung into action to study the new variants to understand how infectious they are, and whether the emergency-authorized vaccines will be effective in protecting against them. Importantly, the Pfizer/BioNTech vaccine has been authorized for 16 years of age and above while Moderna's vaccine is approved for 18 years and above, respectively (Shaman and Galanti 2020[10]; ClinicalTrials.gov/ NCT04368728; BioNTech SE, Pfizer, 2020[1]). However, the new mutant strains bewilderedly rage the population group of younger ages, as opposed to the original strain of SARS-CoV-2. Even though it is hopeful that scientists will proffer a quicker solution to the new variants, however, for now, it is unpredictable whether the new variants will not end up evading vaccine protection.

There have been debates over the SARS-CoV-2 mutations and why these may not affect COVID-19 vaccines when they are readily available. Some proponent views argue that SARS-CoV-2 appears to have a slower rate of mutation than other RNA viruses (Shaman and Galanti 2020[10]). The viewpoint shows that SARS-CoV-2 possesses genetic advantages with proofing mechanisms that help the virus to correct any mismatched nucleotides during genome replication and transcription. Biologically speaking, mutations occur infrequently in some of the critical and essential viral genetic regions. In SARS-CoV-2, one of these regions produces the spike proteins - a structure employed for host binding (Yoshimoto, 2020[14]). For that reason, targeting this protein in the COVID-19 vaccine design may have a clinical advantage to develop a protective immune response as a possible favorable outcome in the quest for an effective vaccine. Unfortunately, attention has been drawn to recently repeated mutations and emerging variants or lineages of SARS-CoV-2 in some regions associated with the spike protein, whereas many vaccine clinical trials have their technology designs based on the knowledge of this protein. There is still a big concern about a potential combination of the evolving mutations in the spike protein and the possibility that this mechanism may help the new strains evade post-vaccination immunity.

**Figure 1:**
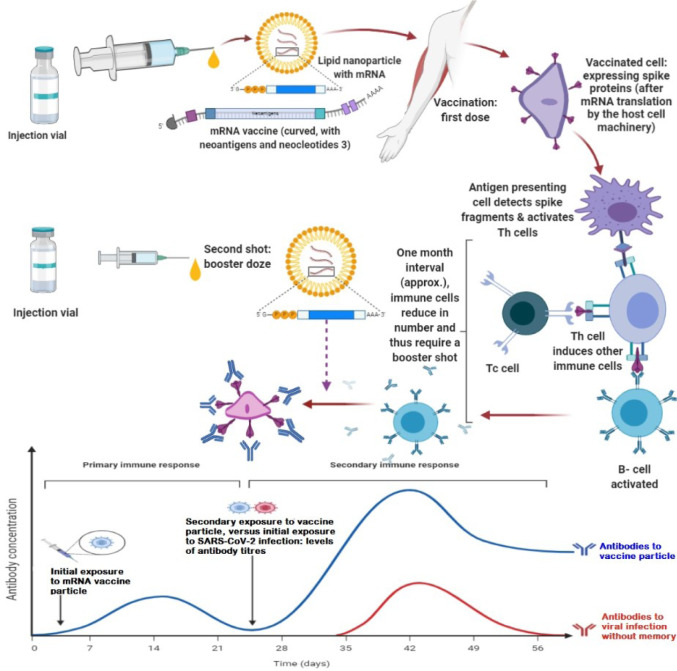
Mechanism showing how mRNA vaccine induces immune response

Meanwhile, the mRNA technology behind the Pfizer-BioNTech's and Moderna's vaccines was anticipated to address the mutation hurdle. These vaccines are designed in such a way that the mRNA mimics the prominent immune dominant part of the virus (Walsh et al., 2020[11]). Mechanistically, when the injected mRNA encounters the host ribosomes, it is interpreted as a transcript from the host DNA and thus translated into an amino acid sequence, which looks exactly like the immunodominant part of the SARS-CoV-2 spike protein. In the meantime, the host DNA is not exposed in this mRNA coding process to produce part of the S-protein. In the clinic, the goal of vaccinating individuals with BNT162b2 is to elicit a strong immune response that would produce relatively long-lasting high affinity neutralizing antibodies (anti-S1 and anti-RBD) that can block the S-protein and the associated RBD interaction with host cell receptors. In addition to neutralizing effect, the immune response is also expected to prompt opsonization-mediated SARS-CoV-2 clearance. In this process, a redundant antibody synthesis could be avoided. As a combined immunoreactive response, immunization with Pfizer-BioNTech's candidate BNT162b2 would also elicit a T-cell immune response of the Th1 type and expected to complement the B-cell response that secrets the S-specific antibodies, and the Tc (cytotoxic T-cells) which kill virus-infected cells (Figure 1(Fig. 1)). At this juncture, it is important to note that immunological memory is an important aspect of the mechanistic process of gaining effective immunization. However, whether the rapidly evolving mutations of SARS-CoV-2 will one day alter the antigenic determinant site or the proteins/carbohydrate surface on the virus is still an issue of time-watch.

## Ethical approval

Not applicable.

## Conflict of interest

None.
